# A decoy set for the thermostable subdomain from chicken villin headpiece, comparison of different free energy estimators

**DOI:** 10.1186/1471-2105-6-301

**Published:** 2005-12-14

**Authors:** Federico Fogolari, Silvio CE Tosatto, Giorgio Colombo

**Affiliations:** 1 Dipartimento di Scienze e Tecnologie Biomediche, Università di Udine, P.le Kolbe 4, 33100 Udine, Italy; 2 Dipartimento di Biologia and CRIBI Biotech Centre, Università di Padova, Viale G. Colombo 3, 35131 Padova, Italy; 3 Istituto di Chimica del Riconoscimento Molecolare, CNR, Via Mario Bianco 9, 20131 Milano, Italy

## Abstract

**Background:**

Estimators of free energies are routinely used to judge the quality of protein structural models. As these estimators still present inaccuracies, they are frequently evaluated by discriminating native or native-like conformations from large ensembles of so-called decoy structures.

**Results:**

A decoy set is obtained from snapshots taken from 5 long (100 ns) molecular dynamics (MD) simulations of the thermostable subdomain from chicken villin headpiece.

An evaluation of the energy of the decoys is given using: i) a residue based contact potential supplemented by a term for the quality of dihedral angles; ii) a recently introduced combination of four statistical scoring functions for model quality estimation (FRST); iii) molecular mechanics with solvation energy estimated either according to the generalized Born surface area (GBSA) or iv) the Poisson-Boltzmann surface area (PBSA) method.

**Conclusion:**

The decoy set presented here has the following features which make it attractive for testing energy scoring functions:

1) it covers a broad range of RMSD values (from less than 2.0 Å to more than 12 Å);

2) it has been obtained from molecular dynamics trajectories, starting from different non-native-like conformations which have diverse behaviour, with secondary structure elements correctly or incorrectly formed, and in one case folding to a native-like structure. This allows not only for scoring of static structures, but also for studying, using free energy estimators, the kinetics of folding;

3) all structures have been obtained from accurate MD simulations in explicit solvent and after molecular mechanics (MM) energy minimization using an implicit solvent method. The quality of the covalent structure therefore does not suffer from steric or covalent problems.

The statistical and physical effective energy functions tested on the set behave differently when native simulation snapshots are included or not in the set and when averaging over the trajectory is performed.

## Background

The correct estimation of the free energy of protein conformations has been the focus of intense research for over 20 years. Despite recent improvements, this goal remains elusive. One important consequence of this is the limited ability to judge the quality of protein models built by homology or "ab initio". In particular, the estimators need to be accurate enough to help in the selection of the most native-like model from an ensemble of closely related alternative conformations. This has been recently identified as one of the bottlenecks limiting the quality and usefulness of protein structure prediction [1].

Free energy estimators can be broadly divided in two different classes: physical effective energy functions based on physical potentials and database derived statistical effective energy functions. Physical effective energy functions (PEEFs) for discrimination of native structures [2-5] generally yield results comparable to statistical effective energy functions (SEEFs) based on the Boltzmann hypothesis [6]. SEEFs are commonly formalized as sets of pairwise potentials of mean force [7-12].

An intuitive test for a free energy estimator is the recognition of the native structure among a large number of well-constructed decoy structures. A number of standard decoy sets, consisting of the native structure plus a large ensemble of more or less incorrect models resembling real protein structures, have been established for benchmarking purposes [13]. The method used for constructing the decoys, which are generally minimized using a simple energy function, introduces some systematic bias. It is therefore important to benchmark a scoring function on different decoy sets in order to assess its generality. As the results for any decoy set are known beforehand, this test does not prevent the scoring function from learning some hidden characteristics shared by all decoy structures, thus limiting its usefulness for practical purposes.

The presently available decoy sets are mainly, if not exclusively, built from "ab initio" models and contain structures which do not cover the full range of root mean square deviation (RMSD) values, with respect to native structure. Most structures, in particular for the older decoy sets, have high RMSD values and are obviously misfolded, limiting the usefulness of the decoy sets. Moreover the simple energy functions used during decoy construction could also account for some systematic bias. For this reason, we have decided to derive a new pool of decoy structures from the snapshots of different trajectories from molecular dynamics simulations of villin headpiece thermostable domain. This new decoy set is evaluated using a battery of PEEFs and SEEFs to give a comparison of their relative performance.

Villin headpiece thermostable domain is a small helical domain, folding on a microsecond time scale. For its limited size, its stability and for fast kinetics of folding it has served as a model for many theoretical studies. All-atom molecular dynamics simulations of the villin headpiece thermostable domain have been performed by several groups using explicit solvent [14-16] and implicit solvent [17-20]. Although simulations were able to produce native-like conformations, the ability of free energy estimators to recognize native structures among all conformations seems rather limited. In a pioneering work Kollman and coworkers were able to identify structures close to native based on MM/PBSA energy [21]. Although molecular mechanics/implicit solvent methods are able to consistently assign low energy to native-like conformations [19,20], their performance depends much on the force field used, the protocol applied and the solvation model adopted. In this context, the well-constructed native- and non-native-like decoys described in this paper will provide a further benchmark for refinement of free energy estimators.

Since this work was started X-ray structures for mutants of the villin headpiece thermostable domain have been deposited in the Protein Data Bank with pdb id.: 1yrf, 1yri, 1wy3, 1wy4 [22]. The RMSD of the trace of these newly deposited structures with respect to the average NMR structure used in the present work (pdb id. 1vii) is 1.92 to 2.09 Å. In general, the results discussed hereafter are essentially the same when any of the other structures deposited in the PDB is used as a reference. Although there are differences among the NMR and X-ray structures [22] all conclusions based on ranking of models according to C_*α *_trace RMSD from native are not significantly affected.

## Results and discussion

### Description of the decoy set

**Figure 1:**
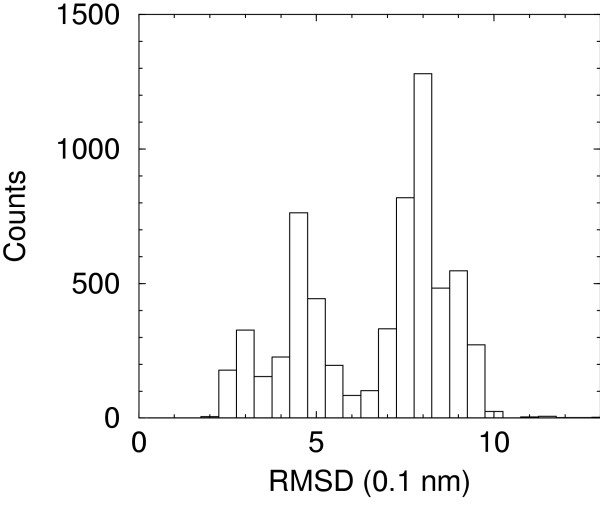
Histogram of RMSD from native structure of the decoy set.

Snapshots from the five long time molecular dynamics trajectories described by De Mori et al. [15,16], extended to 100 ns here, provide a set of conformations which span a large range of RMSDs from native structures. The histogram of counts for each interval of 0.5 Å is reported in Figure 1.

**Figure 2:**
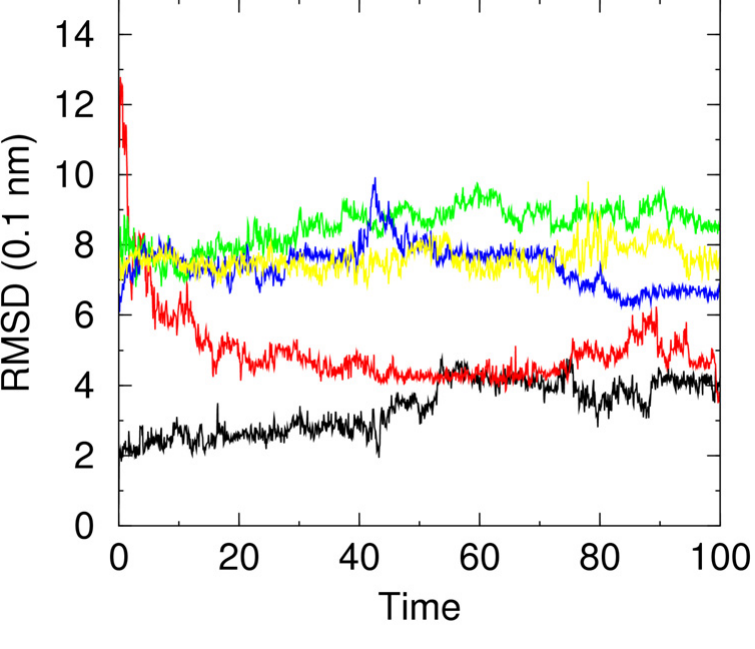
RMSD from native structure, computed on all C_*α *_atoms, versus time (ns) for simulations NATIVE (black), F1 (red), F3 (green), F4 (blue), F7 (yellow).

**Figure 3:**
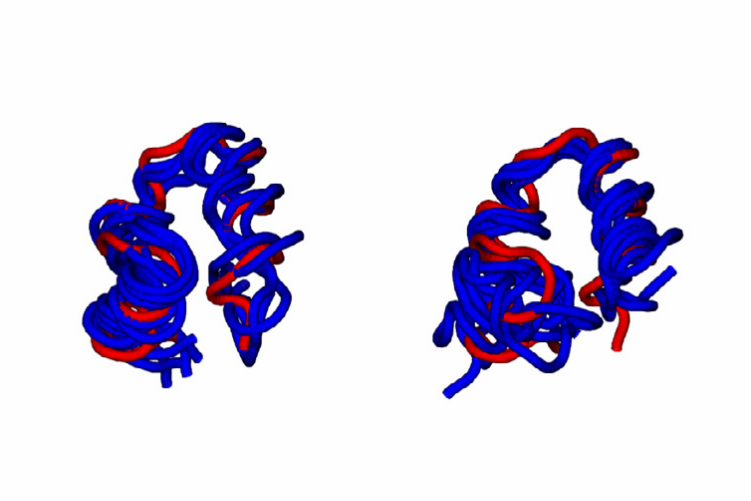
Snapshots taken at 20 ns intervals from NATIVE (left) and F1 (right) simulations superimposed on native structure (pdb id. 1vii).

Although the distribution is not uniform, samples are present for native- and non-native-like conformations. Moreover, in one of the simulations (F1) the peptide assumes a native-like conformation starting from a rather different conformation (see Figure 2). Snapshots taken at 20 ns intervals from NATIVE and F1 simulations have been superimposed on native structure in Figure 3. Superposition of NATIVE snapshots on the starting native structure sets the overall limits for definining native-like conformations (see later). It is evident from superposition of F1 snapshots on native structure that this set qualifies for being defined native-like.

The average radius of gyration of models in each F1, F3, F4 and F7 sets ranges from 9.58 Å (F4) to 10.43 Å (F3). These values are comparable with the average radius of gyration of models in the NATIVE set (9.55 Å). All sets display, after initial collapse at the beginning of the simulation, rather compact structures, as witnessed also by the average solvent accessible surface areas (SASA) ranging from 3013 Å^2 ^(NATIVE) to 3216 Å^2 ^(F3).

The breakdown of SASA in hydrophobic and polar contributions displays a more diverse behavior. NATIVE set has the lowest hydrophobic SASA and the lowest ratio of hydrophobic to polar SASA. Among other sets F1 has the lowest hydrophobic SASA, while sets F4 and F7 show the highest ratio of hydrophobic to polar SASA.

The average number of residues involved in helical conformations has been also monitored for all sets. For NATIVE simulation the average number of residues in *α*- or 3_10_-helices is dropping between 400 ns and 700 ns to reach an average value of ca. 14. This number is comparable to the average number of helical residues in F1 and F3, 13.7 and 13.0 respectively, but definitely higher than the value of 5.4 and 0.3 for F4 and F7, respectively. The average number of hydrogen bonds, however, is not very different among the sets, ranging from 18.5 for F7 to 23.8 for NATIVE.

The quality of structures can be assessed by subjecting the minimized structures to the program Procheck [23] which summarizes the analysis using so-called G-Factors for the quality of dihedral angles, covalent geometry and overall quality of the structure.

Few structures (6 out of 6255) had very high G-Factors which appeared artifactual. These G-factors were reassigned as the average of the previous and next value in the time series. The average overall G-Factor is -0.137, -0.146, -0.183, -0.304, -0.375 for the minimized structures of simulations NATIVE, F1, F3, F4 and F7, respectively. The average G-Factor for dihedral angles is -0.29, -0.30, -0.35, -0.54, -0.64 for the minimized structures of simulations NATIVE, F1, F3, F4 and F7, respectively. The average number of residues in generously allowed or disallowed regions is 0.52, 0.94, 0.59, 1.28, 3.02 for simulations NATIVE, F1, F3, F4 and F7, respectively.

It should be noted that conformations from NATIVE simulation and F1 simulation, folding to a native-like conformation, have higher (i.e. better) overall and dihedral G-Factors than (non-native) conformations from simulations F3, F4, F7. This confirms that statistics of model dihedral angles and covalent structure may be an important value for quality model assessment as recently demonstrated [24-26].

The data discussed in this section are summarized in Table 1.

**Table 1:** Features of the various MD runs. Summary of the main features of the various MD runs. Columns list the average total solvent accessible surface area (Total SASA), its hydrophobic (H-SASA) and polar component (P-SASA), the average radius of gyration (r. gyr.), the average number of residues in *α*- or 3_10_-helices (hel. res.), the average number of hydrogen bonds (H-bonds) and the overall G-Factor from Procheck (Procheck).

Simulation	Total SASA	H-SASA	P-SASA	r. gyr.	hel. res.	H-bonds	Procheck
NATIVE	2990.81	1606.68	1384.12	9.55	17.39	23.8	0.14
F1	3072.17	1712.89	1359.28	9.74	13.69	24.1	0.15
F3	3193.06	1798.77	1394.29	10.43	13.04	24.1	0.18
F4	3117.71	1815.73	1301.98	9.58	5.45	20.9	0.30
F7	3088.72	1825.99	1262.72	10.07	0.29	18.5	0.38

In the following we examine the behaviour of SEEFs and PEEFs on pooled sets of conformations. We consider separately the set containing NATIVE snapshots and the set not containing NATIVE snapshots. The latter set does not contain any information about the native structure. It is homogeneous, in that all snapshots are obtained using the same procedure and it is representative of a realistic prediction scenario. On the other hand, including snapshots from NATIVE simulation allows one to access a lower range of RMSDs from native, which is not available with snapshots from the other simulations.

The decoys arranged in five sets corresponding to the different molecular dynamics trajectories are available in the Internet (see Materials and Methods section).

### Statistical effective energy functions

#### Residue-residue mean contact energy

The contact energy computed according to the methodology of Berrera et al. [10] has been supplemented with a scoring function for the overall quality of the structure (i.e. the G-Factors given by Procheck). The resulting potential allows to separate NATIVE and native-like F1 snapshots from non-native snapshots for most of the conformations.

G-Factors (or any equivalent scoring of the quality of the covalent structure of the protein) are necessary in order to take into account other factors which may raise the energy of a molecule. A few of these, like steric clashes and bad covalent geometry should be removed by molecular dynamics simulation and energy minimization, as performed here. On the contrary, unusual torsion angles are not necessarily removed both for the limited simulation time and for possible inaccuracies in available force fields.

The running average of the contact energy for simulations NATIVE and F1 is mostly separated from that of the other simulations, although the structure is rather flexible and fluctuations in the structure imply fluctuations in energy and in RMSD. Based on this potential (without averaging) a structure at RMSD of 4.0 Å from native could be picked up as the lowest energy structure out of the four simulations F1, F3, F4 and F7.

**Figure 4:**
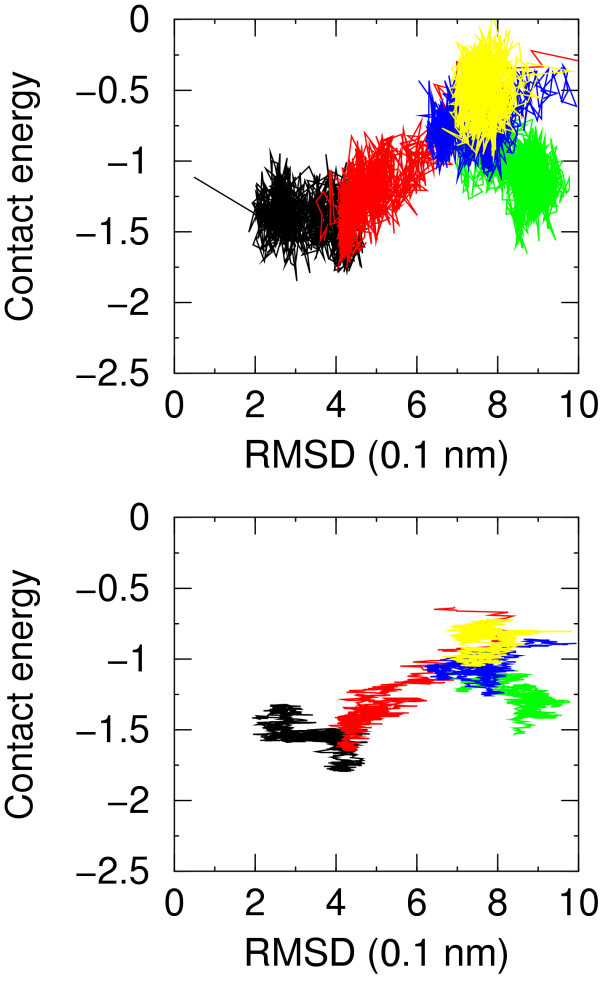
Residue-residue contact energy for simulations NATIVE (black), F1 (red), F3 (green), F4 (blue), F7 (yellow) versus RMSD from native structure. In the upper plot no averaging is applied, in the lower plot a moving average is applied over a window of 50 molecular dynamics snapshots.

Another way of looking at these results is to plot the energy versus the RMSD from native. This is done in Figure 4 where the different simulations are roughly separated. The correlation between contact energy (supplemented by the overall Procheck G-Factor) and RMSD is apparent, although it is hidden by fluctuations. The appearance of the plot greatly improves when the running average of the contact energy is plotted against the RMSD of the structure at the center of the averaging window (Figure 4). This shows that even for such coarse grained potential averaging of fluctuations greatly improves the performance of the scoring function.

The contact energy is strongly correlated with the hydrophobic SASA. The correlation coefficient is 0.67 much larger than the same coefficient for the polar SASA which is -0.40. A similar positive correlation coefficient is found also within each different set of models, showing that this correlation is not artifactual (Table 2). Although this was somehow expected based on the table of pairwise residue-residue contact energies favoring contacts between hydrophobic resiudes, this correlation shows that a simple criterion like burial of hydrophobic SASA may be an effective feature in model quality assessment.

**Table 2:** Energy – feature correlation coefficients. Correlation between energy and selected features of the sets. Correlation coefficients are listed for the sum of contact energy and overall G-Factor from Procheck (contact + pc), the FRST energy (FRST), the molecular mechanics energy (MM), molecular mechanics/generalized Born surface area energy (MM/GBSA) and molecular mechanics/Poisson-Boltzmann surface area energy (MM/PBSA). Selected features are: hydrophobic solvent accessible surface area (H-SASA), radius of gyration (r. gyr.), number of residues in *α*- or 3_10_-helices (hel. res.), number of hydrogen bonds (n. H-bonds). The upper table reports correlation coefficients for the pooled set. The lower table reports the correlation coefficient averaged over the sets NATIVE, F1, F3, F4, F7.

Energy	H-SASA	r. gyr.	hel. res.	H-bonds
contact + pc	0.671	0.305	-0.771	0.575
FRST	0.402	0.014	-0.797	0.597
MM	0.365	0.282	-0.197	0.074
MM/GBSA	0.618	0.273	-0.756	0.535
MM/PBSA	0.719	0.460	-0.621	0.421

Energy	H-SASA	r. gyr.	hel. res.	H-bonds

contact + pc	0.466 ± 0.162	0.340 ± 0.213	0.077 ± 0.203	-0.051 ± 0.180
FRST	-0.065 ± 0.115	-0.092 ± 0.126	-0.097 ± 0.180	0.016 ± 0.106
MM	0.278 ± 0.103	0.488 ± 0.088	-0.062 ± 0.097	-0.020 ± 0.116
MM/GBSA	0.290 ± 0.140	0.256 ± 0.152	0.019 ± 0.248	0.005 ± 0.088
MM/PBSA	0.533 ± 0.129	0.548 ± 0.148	0.078 ± 0.185	-0.056 ± 0.071

The correlation with helical content is less pronounced and largely artifactual because sets NATIVE, F1 and F3, which have the lowest contact energy are also similar in helical content and quite different from sets F4 and F7. When single sets are considered the correlation vanishes.

#### FRST

**Figure 5:**
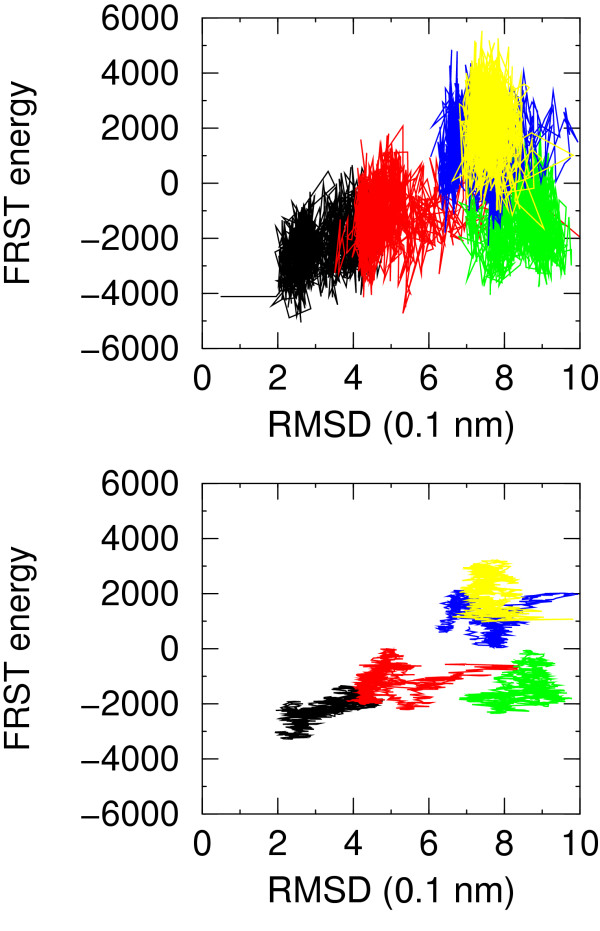
FRST energy for simulations NATIVE (black), F1 (red), F3 (green), F4 (blue), F7 (yellow) versus RMSD from native structure. In the upper plot no averaging is applied, in the lower plot a moving average is applied over a window of 50 molecular dynamics snapshots.

The most striking feature of the FRST energy appears to be the high energy fluctuation between closely related structures. This behaviour, which is more similar to PEEFs, is induced by the torsion angle term of the FRST function. In order to pass from one local minimum to another, it appears necessary to overcome an energy barrier formed by distorsions in local geometry. This effect accounts for the better discrimination of the most native-like conformations in Figure 5. On the other hand it creates some noise in the energy evaluations for the simulations F1, F3 and F4 in particular where FRST has a slight preference for some higher RMSD structures. Overall this SEEF is reliable at discriminating native-like conformations when at least some are present, but it is not as effective at choosing the less erroneous between those with high RMSD.

FRST energy is not correlated with the sets features selected in Table 2, due to the above mentioned fluctuations in the energy. However, it is worth noting that the RAPDF component of FRST energy correlates well with the more coarse grained residue-residue contact energy (correlation coefficient 0.8).

### Physical effective energy functions

#### Molecular mechanics (MM) energy

It is instructive to consider the molecular mechanics energy because it shows how important solvation effects are. With no consideration of solvation effects the energies for all simulations are comparable. The plot of MM energy versus RMSD from native structures (averaged over a window of 50 snapshots) shows no correlation. The best model that could be picked up from the sets of simulations F1, F3, F4, F7 has 7.4 Å RMSD from native. When the average energies are considered, the best model has 8.6 Å RMSD from native. Fluctuations here are rather large, in the range of 100 kcal/mol. No native-like conformation from simulation F1 can be distinguished based on MM energy, as it could be expected.

#### Molecular mechanics/Poisson-Boltzmann surface area (MM/PBSA) energy and molecular mechanics/Generalized Born surface area (MM/GBSA) energy

The performance of the scoring function drastically improves when solvation effects are added to the MM energy function either through the Poisson-Boltzmann model or through the Generalized Born solvent accessibility model. It is apparent that a procedure for smoothing fluctuations should be employed for better reliability. We have previously used the colony energy approach proposed by Honig and co-workers [27] coupled with MM/PBSA energy estimation [5] and we showed that it could greatly improve the energy vs. RMSD curve for a small number of decoy sets. The peculiar nature of the structural models described here makes a simple running average procedure effective.

**Figure 6:**
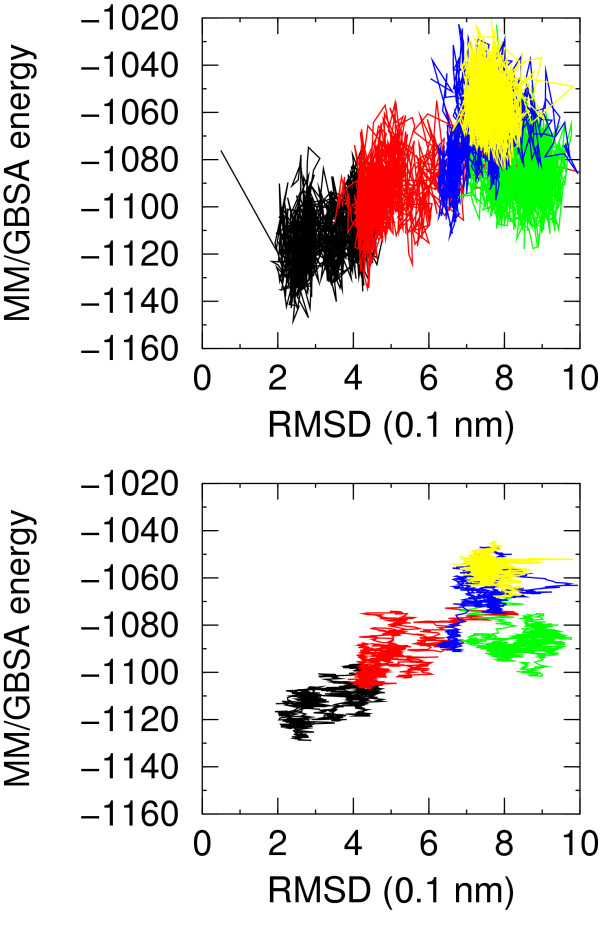
Molecular mechanics/generalized Born surface area energy for simulations NATIVE (black), F1 (red), F3 (green), F4 (blue), F7 (yellow) versus RMSD from native structure. In the upper plot no averaging is applied, in the lower plot a moving average is applied over a window of 50 molecular dynamics snapshots.

**Figure 7:**
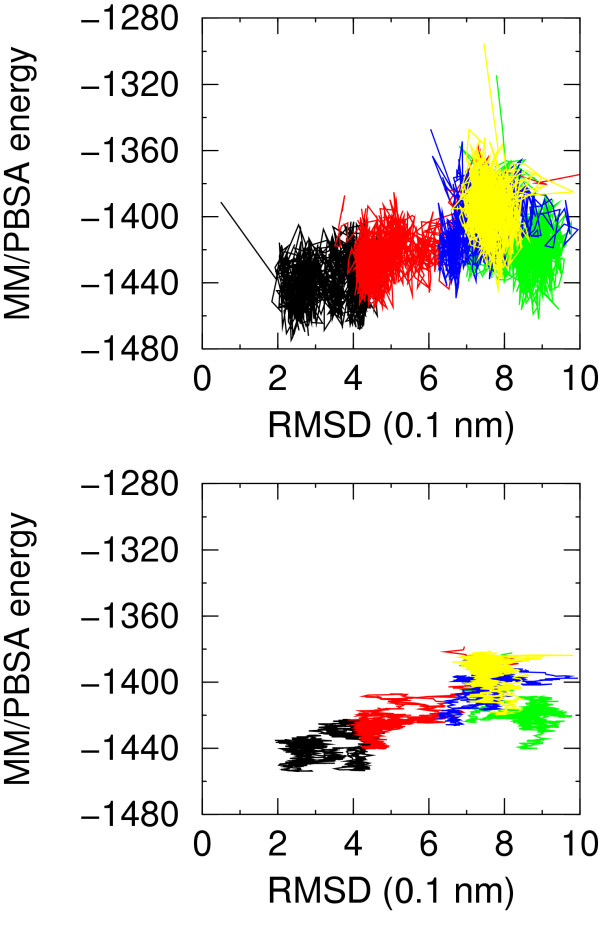
Molecular mechanics/Poisson Boltzmann surface area energy for simulations NATIVE (black), F1 (red), F3 (green), F4 (blue), F7 (yellow) versus RMSD from native structure. In the upper plot no averaging is applied, in the lower plot a moving average is applied over a window of 50 molecular dynamics snapshots.

The MM/GBSA energy for NATIVE and folding simulation F1 is lower than for non-native simulations in a consistent portion of time. The plots of MM/PBSA and MM/GBSA energy versus RMSD from native structure display a very good correlation. This feature is better displayed in the MM/GBSA energy versus RMSD plot (see Figure 6) rather than in the MM/PBSA plot (Figure 7). This is most probably due to the fact that the: i) structures are optimized using MM/GBSA energy and changing the solvation model for energy evaluation may introduce noise; ii) the MM/GBSA method does not suffer from all the numerical approximations implied by the numerical finite difference solution of the Poisson-Boltzmann equation. In this case the best model that could be picked up, based on the energy running average, from simulations starting from non native conformations has a RMSD from native structure of 4.8 Å and 4.4 Å for MM/PBSA and MM/GBSA energy model, respectively. It is worth noting that non-native models in the set F3 have even lower MM/PBSA energy than native-like models of set F1. The running average favors native-like models by few tenths of kcal/mol, largely within the numerical inaccuracies of the methodology. Both MM/GBSA and MM/PBSA energies anticorrelate with the burial of hydrophobic SASA. The correlation with the number of helical residues and number of hydrogen bonds is artifactual, as discussed above, and disappears when single sets are considered.

### Comparison of different effective energy functions

For the present decoy set native-like conformations may be conveniently defined in terms of the RMSD from native structure. The long time simulation of the native structure has an average RMSD compared to the deposited structure of 3.4 Å ± 0.7 Å with a maximum RMSD of 4.8 Å. The latter could be arbitrarily defined as the RMSD for considering a structure native-like.

Tables 3 and 4 report the performance of the SEEFs and PEEFs tested, evaluated with different criteria and according to the RMSD from native of the lowest energy model. Two sets, alternatively including or not snapshots from native simulation, are considered and the energy or scoring function values are considered as they are or averaged over 50 continuous snapshots.

**Table 3:** Evaluation of free energy estimators (native trajectory included). Performance of SEEFs and PEEFs on the set including native simulation. Performance is evaluated according to different methods (see Methods section). The lower part of the table refers to scores averaged over a moving window of 50 molecular dynamics snapshots.

NATIVE+F1+F3+F4+F7 – no average					
Score funct.	logP(B_1_)	logP(B_10_)	F.E.	corr. coeff.	RMSD (Å) best score

contact + pc	-1.08	-1.08	13.8	0.62	3.03
FRST	-1.38	-1.94	23.2	0.48	2.61
MM	-0.25	-1.39	10.6	0.21	7.45
MM/GBSA	-1.71	-2.02	29.6	0.66	2.40
MM/PBSA	-1.79	-2.02	23.2	0.58	2.35

NATIVE+F1+F3+F4+F7 – with average					

Score funct.	logP(B_1_)	logP(B_10_)	F.E.	corr. coeff.	RMSD (Å) best score

contact + pc	-0.91	-0.91	16.3	0.65	3.77
FRST	-1.79	-2.78	43.2	0.54	2.39
MM	-0.70	-1.61	14.8	0.25	4.25
MM/GBSA	-1.14	-1.67	36.1	0.72	2.88
MM/PBSA	-1.54	-2.12	28.6	0.67	2.52

**Table 4:** Evaluation of free energy estimators (native trajectory not included). Performance of SEEFs and PEEFs on the set not including native simulation. Performance is evaluated according to different methods (see Methods section). The lower part of the table refers to scores averaged over a moving window of 50 molecular dynamics snapshots.

F1+F3+F4+F7 – no average					
Score funct.	logP(B_1_)	logP(B_10_)	F.E.	corr. coeff.	RMSD (Å) best score

contact + pc	-2.55	-2.55	29.8	0.40	4.03
FRST	-0.70	-1.44	11.9	0.17	5.31
MM	-0.34	-0.64	2.3	-0.07	7.45
MM/GBSA	-1.20	-1.70	23.2	0.38	4.37
MM/PBSA	-0.04	-1.02	15.1	0.32	8.90

F1+F3+F4+F7 – with average					

Score funct.	logP(B_1_)	logP(B_10_)	F.E.	corr. coeff.	RMSD (Å) best score

contact + pc	-1.11	-1.61	39.2	0.43	4.43
FRST	-0.15	-0.31	10.7	0.21	7.91
MM	-0.07	-0.08	0.0	-0.14	8.59
MM/GBSA	-1.20	-1.83	34.5	0.44	4.37
MM/PBSA	-0.85	-1.23	24.6	0.38	4.80

For the set with native simulation snapshots included, averaging improves significantly the performance of the FRST scoring function and MM energy function. For the set not including native simulation snapshots, averaging improves significantly the performance of MM/GBSA and MM/PBSA energy functions which are known to be affected by large fluctuations (see the previous sections).

For the set including native simulation snapshots the FRST scoring function, MM/GBSA and MM/PBSA energy functions show the best performance and they all recognize native-like models with RMSD from native less than 3.0 Å.

The poor performance of MM energy points out the need to consider solvation effects. For the set not including native simulation snapshots SEEFs appear superior to PEEFs. Averaging makes MM/GBSA and MM/PBSA more effective while it worsens the performance of atom-based FRST scoring function. The sensitivity of FRST to molecular details is greater than that of the residue-based contact energy [10] for which, indeed, averaging has an overall beneficial effect.

Since FRST is trained on native structures it is likely that while assigning low scores to native features, it assigns exceedingly bad scores to non-native-like features. This would explain the effect of averaging the score over closely related structures. Overall from the set not including native simulation snapshots it is seen that most methods are able to detect models with reasonably low RMSD from native (4.0 Å to 5.3 Å).

## Conclusion

The decoy set presented here displays features which make it attractive for testing energy scoring functions:

1) it covers a broad range of RMSD values (from less than 2.0 Å to more than 12.0 Å);

2) it has been obtained from molecular dynamics trajectories, starting from native and different non-native conformations which have diverse behaviour, with secondary structure elements correctly or incorrectly formed, and in one case folding to a native-like structure. This allows not only for scoring of static structures, but also for studying, using free energy estimators, the kinetics of folding;

3) All structures have been obtained from accurate MD simulations in explicit solvent and after molecular mechanics (MM) energy minimization using an implicit solvent method. The quality of the covalent structure therefore does not suffer from major steric or covalent problems.

Different free energy or scoring functions have been tested on the sets. The performance of SEEFs and PEEFs depends strongly on the decoy set. Here we have a decoy set with native, native-like and non-native structures. The set not including decoys from the native trajectory is representative of a realistic prediction scenario. Moreover a simple running averaging procedure can be employed in order to smooth free energy fluctuations, because decoys are obtained by sequential sampling of molecular dynamics trajectory. These features are not common for decoy sets.

The performance of free energy estimators may be assessed therefore in different contexts: 1) when snapshots from native simulation are included in the set; 2) when only snapshots from simulations starting from non-native conformations are included in the set.

Moreover the effect of averaging may be easily assessed.

When native structures are included in the set, i.e., when the set includes models very close to native structure, atom based energy functions perform very well. The atom-based FRST SEEF compares well with atom-based MM/PBSA and MM/GBSA PEEFs.

When snapshots from native simulation are not included in the set, the performance of residue based SEEF less sensitive to molecular details improves over atom-based SEEF and PEEFs. For MM/GBSA and MM/PBSA averaging has a beneficial effect being able to smooth large fluctuations expected in the methodology. The effect of averaging on FRST SEEF is dominated by relatively poor discriminative power on non-native free energy.

Overall these results suggest that different free energy estimators, possibly in a hierarchical scheme, should be used depending on the expected quality of predictive models. Application of some form of averaging in order to smooth fluctuations due to molecular details, should be considered carefully depending on the energy function employed.

## Methods

### Molecular Dynamics simulations and analysis

The structures and trajectories used in this work were selected as described in a previous paper [15,16], which we briefly outline here. Seven uncorrelated conformations were chosen from a coarse-grained Monte Carlo (MC) trajectory thermalised at the transition temperature *T*_*c*_. The MC evolution occurs in a simplified free-energy landscape in which an off-lattice representation of the protein with every residue represented by a bead is used to allow an efficient selection of marginally-compact structures, which are subsequently taken as viable initial conformations for the subsequent all-atom MD. The coarse-grained MC structural representation is connected to the one with atomic resolution through a "fine-graining" reconstruction algorithm described in De Mori et al., 2005 [16]. Starting from different reconstructed MC structures, seven all-atom simulations (F1, F2, ... F7) were carried out. We have concentrated on four of them, whose length was extended to 100 ns, namely F1, F3, F4 and F7, which examplify the variety of observed dynamical behavior. To obtain a reference trajectory, a 100 ns long simulation was run starting from the NOE derived minimized average structure (pdb code 1VII.pdb). This trajectory is labeled NATIVE throughout the text. Every all-atom model obtained was subjected to Molecular Dynamics simulations (simulations NATIVE, F1, F3, F4, F7), using the same simulation protocol and conditions. All basic NH_2 _groups were considered protonated while all the acidic COO^- ^groups were considered deprotonated. These conditions resulted in a total charge of +2 on the protein. The protein in each simulation was solvated with explicit water molecules in a periodic octahedral box large enough to contain the protein and 1.0 nm of solvent on all sides. The simple point charge (SPC) [28] water model was used to simulate the solvent. Electroneutrality of the system was ensured by the addition of two negatively charged chloride ions.

The same equilibration protocol was used in all simulation:

**1. **Each system was initially energy minimized with a steepest descent method for 1000 steps. The calculation of electrostatic forces utilized the PME implementation of the Ewald summation method [29]. The GROMOS96 force field [30,31] was used.

**2. **To release excess strain in simulations F1, F3, F4, F7 due to possible artifacts in the reconstruction procedure, three sequential MD runs of 50 ps each with position restraints on the protein, backbone and sidechains, respectively, were performed. In the first, the protein atoms were kept fixed with the solvent molecules free to move. In the subsequent run, the backbone of the protein was kept fixed with both the sidechains and solvent free to move in MD. In the third and final simulation, the sidechains were kept fixed, while the backbone and solvent atoms were allowed to move.

**3. **After these first relaxation steps, each system was heated to 300 K by 200 ps of MD simulation under NPT conditions, with no restraints, by weak coupling to a bath of constant pressure (P_0 _= 1 bar, coupling time *τ*_*P *_= 0.5 ps) [32].

**4. **Each of the systems was then equilibrated by 50 ps of unrestrained MD with coupling to an external temperature bath [32] with coupling constant of 0.1 ps.

**5. **The production runs at 300 K, using NPT conditions were all 100 ns long in the case of the simulation starting from the NMR determined structure 1VII.pdb (NATIVE) and simulations F1, F3, F4, F7.

The number of explicit water molecules and the structural features of the simulations are reported in Table 5. In all production runs, the temperature was maintained close to the intended values, by weak coupling to an external temperature bath [32] with a coupling constant of 0.1 ps. The protein and the rest of the system were coupled separately to the temperature bath. The LINCS algorithm [33] was used to constrain all bond lengths. For the water molecules the SETTLE algorithm [34] was used. A dielectric permittivity, *∊ *= 1, and a time step of 2 fs were used. All atoms were given an initial velocity obtained from a Maxwellian distribution at the desired initial temperature of 300 K.

**Table 5:** Features of the various MD simulations. Summary of the main feature of the various MD runs. Number of solvent particles in each of the simulations. For trajectories F1–F7 are also provided the fraction of local and non-local native contacts at the beginning of the MD run, as well as the initial RMSD over the core of the protein (residues 9–32).

Simulation	Water Molecules	ql0 MathType@MTEF@5@5@+=feaafiart1ev1aaatCvAUfKttLearuWrP9MDH5MBPbIqV92AaeXatLxBI9gBaebbnrfifHhDYfgasaacH8akY=wiFfYdH8Gipec8Eeeu0xXdbba9frFj0=OqFfea0dXdd9vqai=hGuQ8kuc9pgc9s8qqaq=dirpe0xb9q8qiLsFr0=vr0=vr0dc8meaabaqaciaacaGaaeqabaqabeGadaaakeaacqWGXbqCdaqhaaWcbaGaemiBaWgabaGaeGimaadaaaaa@3093@	qnl0 MathType@MTEF@5@5@+=feaafiart1ev1aaatCvAUfKttLearuWrP9MDH5MBPbIqV92AaeXatLxBI9gBaebbnrfifHhDYfgasaacH8akY=wiFfYdH8Gipec8Eeeu0xXdbba9frFj0=OqFfea0dXdd9vqai=hGuQ8kuc9pgc9s8qqaq=dirpe0xb9q8qiLsFr0=vr0=vr0dc8meaabaqaciaacaGaaeqabaqabeGadaaakeaacqWGXbqCdaqhaaWcbaGaemOBa4MaemiBaWgabaGaeGimaadaaaaa@31F8@	initial core RMSD
F1	6568	0.67	0.1	8.4
F3	3892	0.61	0.15	6.1
F4	3140	0.50	0.10	4.5
F7	2911	0.46	0.04	5.5

NATIVE	2768			

All the minimization, MD runs were performed using the GROMACS software package. [35].

### Description of the decoy set

All snapshots from the five trajectories described above have been energy minimized in a two-step fashion using the program CHARMM and the CHARMM forcefield (version 27b2) [36,37]. First all C_*α *_coordinates have been fixed and the energy minimized with 20 steepest descent and 30 conjugate gradients steps with 10 conjugate gradient cycles, in order to remove high energy spots, like van der Waals clashes. During this minimization a distance dependent dielectric constant has been employed (*ε *= 1*r*) with a cutoff on non-bond interactions of 12 Å. In the second step the structure is totally unrestrained and a more accurate solvent model (the Generalized Born model) has been employed using the parameters suggested by Qiu et al. [38], in order to reduce artifacts ensuing from missing solvent. First 50 steepest descent minimization steps have been performed followed by 200 conjugate gradients minimization steps with 10 conjugate gradient cycles. The energy minimized structures have been deposited in the Decoys'R'us database with the name vhp_mcmd (villin headpiece – Monte Carlo molecular dynamics) (, [13]).

### Amino acid empirical contact energy

Residue-residue contact energies have been computed according to the methodology described by Berrera et al. [10]. A contact between two residues exists when these are not adjacent and when a heavy atom of the sidechain of one of the residues is closer than a threshold distance to a heavy atom of the sidechain of the other residue. The threshold was chosen, according to Berrera et al., 2003 [10] as the sum of the van der Waals radii of the two atoms plus 1.0 Å. The table of contact energies of Berrera et al. [10] has been used and the total energy has been divided by the number of aminoacids. In order to take into account the overall quality of each model the overall G-factor (with changed sign) from Procheck has been added to the contact energy.

### FRST

The FRST energy function is a potential covering the main features of native protein structures. It has been fully presented elsewhere [26]. Briefly put, the energy function uses a linear combination of four different statistical potentials to calculate a pseudo-energy which can be used to estimate the quality of a protein structure. The most important feature is the quality of the local geometry of the protein backbone, expressed by a residue-specific torsion angle potential. The (*φ,ψ*) angles of each residue are used to construct a 10 × 10 degree propensity map for each of the twenty amino acids. The RAPDF potential [7] is a statistical pairwise potential used as the second most important feature in the total energy. The remaining two energy terms are a statistical solvation term constructed in analogy to ref. [39] and a simple count of intra-chain backbone hydrogen bonds. The weighting factors were derived from optimizing the results for a training set of 3,757 models submitted during CASP-4, covering the full range from easy comparative modelling all the way to difficult novel folds targets.

### MM energy, MM/GBSA and MM/PBSA free energy

The MM/PBSA methodology, as applied here, has been described at length and its performance has been tested [5,40]. In this methodology the free energy of a system is written as the sum of an intrasolute term, computed according to one of the available molecular mechanics forcefield, and a solvation term which is further split in an electrostatic term and a hydrophobic term. The latter term is taken as proportional to the solvent accessible surface area. The electrostatic solvation term is computed according to the Poisson-Botzmann theoretical framework [41].

In summary, the free energy of the macroscopic state C, unless a constant term and entropic contributions, is:

ΔGC=U(r→1,  ..,r→n)+ΔGPB(r→1,  ..,r→n)+ΔGSA(r→1,  ..,r→n)
 MathType@MTEF@5@5@+=feaafiart1ev1aaatCvAUfKttLearuWrP9MDH5MBPbIqV92AaeXatLxBI9gBaebbnrfifHhDYfgasaacH8akY=wiFfYdH8Gipec8Eeeu0xXdbba9frFj0=OqFfea0dXdd9vqai=hGuQ8kuc9pgc9s8qqaq=dirpe0xb9q8qiLsFr0=vr0=vr0dc8meaabaqaciaacaGaaeqabaqabeGadaaakeaacqqHuoarcqWGhbWrdaahaaWcbeqaaiabdoeadbaakiabg2da9iabdwfavjabcIcaOiqbdkhaYzaalaWaaSbaaSqaaiabigdaXaqabaGccqGGSaalcaaMc8UaaGPaVlabc6caUiabc6caUiabcYcaSiqbdkhaYzaalaWaaSbaaSqaaiabd6gaUbqabaGccqGGPaqkcqGHRaWkcqqHuoarcqWGhbWrdaahaaWcbeqaaiabdcfaqjabdkeacbaakiabcIcaOiqbdkhaYzaalaWaaSbaaSqaaiabigdaXaqabaGccqGGSaalcaaMc8UaaGPaVlabc6caUiabc6caUiabcYcaSiqbdkhaYzaalaWaaSbaaSqaaiabd6gaUbqabaGccqGGPaqkcqGHRaWkcqqHuoarcqWGhbWrdaahaaWcbeqaaiabdofatjabdgeabbaakiabcIcaOiqbdkhaYzaalaWaaSbaaSqaaiabigdaXaqabaGccqGGSaalcaaMc8UaaGPaVlabc6caUiabc6caUiabcYcaSiqbdkhaYzaalaWaaSbaaSqaaiabd6gaUbqabaGccqGGPaqkaaa@6871@

Δ*G*^*PB*^(r→1
 MathType@MTEF@5@5@+=feaafiart1ev1aaatCvAUfKttLearuWrP9MDH5MBPbIqV92AaeXatLxBI9gBaebbnrfifHhDYfgasaacH8akY=wiFfYdH8Gipec8Eeeu0xXdbba9frFj0=OqFfea0dXdd9vqai=hGuQ8kuc9pgc9s8qqaq=dirpe0xb9q8qiLsFr0=vr0=vr0dc8meaabaqaciaacaGaaeqabaqabeGadaaakeaacuWGYbGCgaWcamaaBaaaleaacqaIXaqmaeqaaaaa@2F47@,..,r→n
 MathType@MTEF@5@5@+=feaafiart1ev1aaatCvAUfKttLearuWrP9MDH5MBPbIqV92AaeXatLxBI9gBaebbnrfifHhDYfgasaacH8akY=wiFfYdH8Gipec8Eeeu0xXdbba9frFj0=OqFfea0dXdd9vqai=hGuQ8kuc9pgc9s8qqaq=dirpe0xb9q8qiLsFr0=vr0=vr0dc8meaabaqaciaacaGaaeqabaqabeGadaaakeaacuWGYbGCgaWcamaaBaaaleaacqWGUbGBaeqaaaaa@2FBC@)is the free energy difference computed according to the Poisson-Boltzmann equation for charging the rigid molecule in solution and in gas phase. This electrostatic term has been computed either according to the Poisson-Boltzmann equation or according to the Generalized Born (GB) model [38]. Δ*G*^*SA*^(r→1
 MathType@MTEF@5@5@+=feaafiart1ev1aaatCvAUfKttLearuWrP9MDH5MBPbIqV92AaeXatLxBI9gBaebbnrfifHhDYfgasaacH8akY=wiFfYdH8Gipec8Eeeu0xXdbba9frFj0=OqFfea0dXdd9vqai=hGuQ8kuc9pgc9s8qqaq=dirpe0xb9q8qiLsFr0=vr0=vr0dc8meaabaqaciaacaGaaeqabaqabeGadaaakeaacuWGYbGCgaWcamaaBaaaleaacqaIXaqmaeqaaaaa@2F47@,...,r→n
 MathType@MTEF@5@5@+=feaafiart1ev1aaatCvAUfKttLearuWrP9MDH5MBPbIqV92AaeXatLxBI9gBaebbnrfifHhDYfgasaacH8akY=wiFfYdH8Gipec8Eeeu0xXdbba9frFj0=OqFfea0dXdd9vqai=hGuQ8kuc9pgc9s8qqaq=dirpe0xb9q8qiLsFr0=vr0=vr0dc8meaabaqaciaacaGaaeqabaqabeGadaaakeaacuWGYbGCgaWcamaaBaaaleaacqWGUbGBaeqaaaaa@2FBC@) is the so-called hydrophobic energy and may be taken proportional to the solute-solvent interface area, i.e. Δ*G*^*SA *^= *γA*, where *A *is defined as the area of the solvent accessible surface. There is no agreement in the literature on the proportionality coefficient (i.e. the surface tension coefficient). A value of 50 cal mol^-1 ^Å^-2 ^has been proposed by Nicholls et al. [42] for proteins. This value has been significantly decreased by us for use in MM/PBSA framework because van der Waals intramolecular interactions will account for a large part of this surface tension. Based on simple considerations we have proposed a value of 20 cal mol^-1 ^Å^-2 ^[43].

If we assume that entropic contributions are the same for all sampled conformations, we can use Δ*G*^*c*^ for estimating the stability of each conformation. We have used both the MM energy computed using CHARMM [36,37], the MM/GBSA energy computed using CHARMM and MSMS [44] for solvent accessible surface area and MM/PBSA computed using CHARMM and the program UHBD [45,46]. The linear version of the PB equation has been used as justified by the low charge density of the protein [47]. A procedure using focusing makes the computation of PB energies rather efficient [43,48].

### Averaging over molecular dynamics simulations

The values of MM, MM/GBSA and MM/PBSA energy during a simulation are affected by very large fluctuations, typically in the range of 100 kcal/mol. In order to damp fluctuations, a running average within a window of 50 snapshots contiguous in time was performed and the average energy or score was assigned to the model at the center of the moving window.

### Performance evaluation

The performance of the scoring functions used has been evaluated using the parameters recently proposed and employed by Samudrala and coworkers [11]. We summarize here the definitions and features of the scoring functions employed:

1) the logP(B_1_) is defined as:

logP(B1)=log10(r1N)
 MathType@MTEF@5@5@+=feaafiart1ev1aaatCvAUfKttLearuWrP9MDH5MBPbIqV92AaeXatLxBI9gBaebbnrfifHhDYfgasaacH8akY=wiFfYdH8Gipec8Eeeu0xXdbba9frFj0=OqFfea0dXdd9vqai=hGuQ8kuc9pgc9s8qqaq=dirpe0xb9q8qiLsFr0=vr0=vr0dc8meaabaqaciaacaGaaeqabaqabeGadaaakeaaieGacqWFSbaBcqWFVbWBcqWFNbWzcqWGqbaucqGGOaakcqWGcbGqdaWgaaWcbaGaeGymaedabeaakiabcMcaPiabg2da9iab=XgaSjab=9gaVjab=DgaNnaaBaaaleaacqaIXaqmcqaIWaamaeqaaOGaeiikaGYaaSaaaeaacqWGYbGCdaWgaaWcbaGaeGymaedabeaaaOqaaiabd6eaobaacqGGPaqkaaa@427F@

where *r*_1 _is the rank in RMSD from native for the best scoring model, normalized by the number of decoys in the set.

2) the logP(B_10_) is defined as:

log⁡P(B10)=log⁡10(r110N)
 MathType@MTEF@5@5@+=feaafiart1ev1aaatCvAUfKttLearuWrP9MDH5MBPbIqV92AaeXatLxBI9gBaebbnrfifHhDYfgasaacH8akY=wiFfYdH8Gipec8Eeeu0xXdbba9frFj0=OqFfea0dXdd9vqai=hGuQ8kuc9pgc9s8qqaq=dirpe0xb9q8qiLsFr0=vr0=vr0dc8meaabaqaciaacaGaaeqabaqabeGadaaakeaacyGGSbaBcqGGVbWBcqGGNbWzcqWGqbaucqGGOaakcqWGcbGqdaWgaaWcbaGaeGymaeJaeGimaadabeaakiabcMcaPiabg2da9iGbcYgaSjabc+gaVjabcEgaNnaaBaaaleaacqaIXaqmcqaIWaamaeqaaOGaeiikaGYaaSaaaeaacqWGYbGCdaqhaaWcbaGaeGymaedabaGaeGymaeJaeGimaadaaaGcbaGaemOta4eaaiabcMcaPaaa@4557@

where r110
 MathType@MTEF@5@5@+=feaafiart1ev1aaatCvAUfKttLearuWrP9MDH5MBPbIqV92AaeXatLxBI9gBaebbnrfifHhDYfgasaacH8akY=wiFfYdH8Gipec8Eeeu0xXdbba9frFj0=OqFfea0dXdd9vqai=hGuQ8kuc9pgc9s8qqaq=dirpe0xb9q8qiLsFr0=vr0=vr0dc8meaabaqaciaacaGaaeqabaqabeGadaaakeaacqWGYbGCdaqhaaWcbaGaeGymaedabaGaeGymaeJaeGimaadaaaaa@3114@ is the rank in RMSD from native of the structure with lowest RMSD from native among the ten best scoring models, normalized by the number of decoys in the set.

The values of logP(B_1_) and logP(B_10_) depend on the fraction of models with RMSD from native similar to the best model. These values are the logarithm of the probability of scoring the best model as the lowest or among the ten lowest energy models [11].

3) the fraction enrichment parameter defined as the fraction of the 10% lowest RMSD models which is found among the 10% best scoring models. This parameter provides evidence for the tendency of the scoring system to assign low RMSD models best scores.

4) the correlation coefficient between score and RMSD from native. This parameter may be ineffective if the scoring function is sensitive to minor model details and if it is not accurate in all the range of RMSDs.

5) the RMSD from native of the best score model. This parameter is directly related to the performance of the scoring function in recognizing native-like models.

These five parameters have been considered for the pool of NATIVE, F1, F3, F4 and F7 minimized snapshots and for the same pool not including snapshots from NATIVE simulation. The decoys from the latter ensemble do not include any information on native structure. The same analysis has been repeated taking for each model the average of the energy or scoring function in a window of 50 snapshots.

## Authors' contributions

FF, SCET and GC conceived the project. GC performed the molecular dynamics simulations, their analysis, and provided snapshots, FF developed the MM/GBSA minimization protocol and MM, MM/GBSA, MM/PBSA and contact energy evaluation, SCET developed the FRST evaluation protocol and analysis, FF and SCET designed analysis methods. FF performed the performance and correlation analysis. All authors edited the manuscript.

## References

[B1] Valencia A (2005). Protein refinement: a new challenge for CASP in its 10th anniversary. Bioinformatics.

[B2] Hao M, Scheraga H (1998). Molecular mechanisms for cooperative folding of proteins. J Mol Biol.

[B3] Lazaridis T, Karplus M (1999). Discrimination of the native from misfolded protein models with an energy function including implicit solvation. J Mol Biol.

[B4] Petrey D, Honig B (2000). Free energy determinants of tertiary structure and the evaluation of protein models. Protein Sci.

[B5] Fogolari F, Tosatto S (2005). Application of MM/PBSA colony free energy to loop decoy discrimination: towards correlation between energy and root mean square deviation. Prot Sci.

[B6] Sippl M (1993). Boltzmann's principle, knowledge-based mean fields and protein folding. An approach to the computational determination of protein structures. J Comput Aided Mol Des.

[B7] Samudrala R, Moult J (1998). An all-atom distance-dependent conditional probability discriminatory function for protein structure prediction. J Mol Biol.

[B8] Lu H, Skolnick J (2001). A distance-dependent atomic knowledge-based potential for improved protein structure selection. Proteins.

[B9] Zhou H, Zhou Y (2002). Distance-scaled, finite ideal-gas reference state improves structure-derived potentials of mean force for structure selection and stability prediction. Protein Sci.

[B10] Berrera M, Molinari H, Fogolari F (2003). Amino acid empirical contact energy definitions for fold recognition in the space of contact maps. BMC Bioinformatics.

[B11] Wang K, Fain B, Levitt M, Samudrala R (2004). Improved protein structure selection using decoy-dependent discriminatory functions. BMC Struct Biol.

[B12] Zhang C, Liu S, Zhou H, Zhou Y (2004). An accurate, residue-level, pair potential of mean force for folding and binding based on the distance-scaled, ideal-gas reference state. Protein Sci.

[B13] Samudrala R, Levitt M (2000). Decoys 'R' us: a database of incorrect protein conformations to improve protein structure prediction. Protein Sci.

[B14] Duan Y, Kollman P (1998). Pathways to a protein folding intermediate observed in a 1-microsecond simulation in aqueous solution. Science.

[B15] de Mori G, Micheletti C, Colombo G (2004). All-atom folding simulations of the Villin headpiece from stochastically-selected coarse-grained structures. J Phys Chem B.

[B16] de Mori G, Colombo G, Micheletti C (2005). Study of the Villin headpiece folding dynamics by combining coarse-grained Monte Carlo evolution and all-atom molecular dynamics. Proteins.

[B17] Zagrovic B, Snow C, Shirts M, Pande V (2002). Simulation of folding of a small alpha-helical protein in atomistic detail using worldwide-distributed computing. J Mol Biol.

[B18] Pande V, Baker I, Chapman J, Elmer S, Khaliq S, Rhee Y, Shirts M, Snow C, sorin E, Zagrovic B (2003). Atomistic protein folding simulation on the submillisecond time scale using worldwide distributed computing. Biopolymers.

[B19] Ripoll D, Vila J, Scheraga H (2004). Folding of the villin headpiece subdomain from random structures. Analysis of the charge distribution as a function of pH. J Mol Biol.

[B20] Herges T, Wenzel W (2005). Free energy landscape of the villin headpiece in an all-atom force field. Structure.

[B21] Lee MR, Baker D, Kollman PA (2001). 2.1 and 1.8 Å average C_*α *_RMSD structure predictions on two small proteins, HP-36 and s15. J Am Chem Soc.

[B22] Chiu T, Kubelka J, Herbst-Irmer R, Eaton W, Hofrichter J, Davies D (2005). High-resolution X-Ray crystal structure of the villin headpiece subdomain, an ultrafast folding protein. Proc Natl Acad Sci.

[B23] Laskowski R, MacArthur M, Moss D, Thornton J (1993). PROCHECK: A program to check the stereochemical quality of protein structures. J Appl Cryst.

[B24] Shortle D (2002). Composites of local structure propensities: evidence for local encoding of long range structure. Protein Sci.

[B25] Fang Q, Shortle D (2005). A consistent set of statistical potentials for quantifying local side-chain and backbone interactions. Proteins.

[B26] Tosatto S (2005). The Victor/FRST Function for Model Quality Estimation. J Comput Biol.

[B27] Xiang Z, Soto SC, Honig B (2002). Evaluating free energies: the colony energy and its application to the problem of loop prediction. Proc Natl Acad Sci USA.

[B28] Berendsen HJC, Grigera JR, Straatsma TP (1987). The missing term in effective pair potentials. J Phys Chem.

[B29] Darden T, York D, Pedersen L (1993). Particle Mesh Ewald. An N.log(N) method for Ewald sums in large systems. Journal of Chemical Physics.

[B30] van Gunsteren WF, Daura X, Mark AE (1998). GROMOS Force Field. Encyclopedia of Computational Chemistry.

[B31] van Gunsteren WF, Billeter SR, Eising AA, Hünenberger PH, Krüger P, Mark AE, Scott WRP, Tironi IG (1996). Biomolecular Simulation: The GROMOS96 Manual and User Guide.

[B32] Berendsen HJC, Postma JPM, van Gunsteren WF, Di Nola A, Haak JR (1984). Molecular dynamics with coupling to an external bath. J Chem Phys.

[B33] Hess B, Bekker H, Fraaije JGEM, Berendsen HJC (1997). A linear constraint solver for molecular simulations. J Comp Chem.

[B34] Miyamoto S, Kollman PA (1992). SETTLE: An analytical version of the SHAKE and RATTLE algorithms for rigid water models. J Comp Chem.

[B35] van der Spoel D, van Drunen R, Berendsen HJC (1994). GRoningen MAchine for Chemical Simulations.

[B36] Brooks BR, Bruccoleri RE, Olafson BD, States DJ, Swaminathan S, Karplus M (1983). CHARMM: A Program for Macromolecular Energy Minimization and Dynamics Calculations. J Comput Chem.

[B37] MacKerell ADJ, Bashford D, Bellott M, Dunbrack RLJ, Evanseck JD, Field MJ, Fischer S, Gao J, Guo H, Ha S, Joseph-McCarthy D, Kuchnir L, Kuczera K, Lau FTK, Mattos C, Michnick S, Ngo T, Nguyen DT, Prodhom B, Reiher WEI, Roux B, Schlenkrich M, Smith JC, Stote R, Straub J, Watanabe M, Wiorkiewicz-Kuczera J, Yin D, Karplus M (1998). All-atom empirical potential for molecular modeling and dynamics Studies of proteins. J Phys Chem B.

[B38] Qiu D, Shenkin P, Hollinger F, Still W (1997). The GB/SA Continuum Model for Solvation. A Fast Analytical Method for the Calculation of Approximate Born Radii. J Phys Chem.

[B39] Jones DT (1999). GenTHREADER: an efficient and reliable protein fold recognition method for genomic sequences. J Mol Biol.

[B40] Fogolari F, Moroni E, Wojciechowski M, Baginski M, Ragona L, Molinari H (2005). MM/PBSA analysis of molecular dynamics simulations of bovine b-lactoglobulin: free energy gradients in conformational transitions?. Proteins.

[B41] Fogolari F, Brigo A, Molinari H (2002). The Poisson-Boltzmann equation for biomolecular electrostatics: a tool for structural biology. J Mol Recogn.

[B42] Nicholls A, Sharp KA, Honig B (1991). Protein folding and association: insights from the interfacial and thermodynamic properties of hydrocarbons. Proteins: Struct Funct Genet.

[B43] Fogolari F, Brigo A, Molinari H (2003). Protocol for MM/PBSA molecular dynamics simulations of proteins. Biophys J.

[B44] Sanner M, Spehner JC, Olson A (1996). Reduced surface: an efficient way to compute molecular surfaces. Biopolymers.

[B45] Madura JD, Davis ME, Gilson MK, Wade R, Luty BA, McCammon JA (1994). Biological applications of electrostatics calculations and Brownian dynamics simulations. Rev Comp Chem.

[B46] Madura JD, Briggs JM, Wade R, Davis ME, Luty BA, Ilin A, Antosiewicz JA, Gilson MK, Bagheri B, Ridgway Scott L, McCammon JA (1995). Electrostatics and diffusion of molecules in solution: simulations with the University of Houston Brownian Dynamics program. Comput Commun Phys.

[B47] Fogolari F, Zuccato P, Esposito G, Viglino P (1999). Biomolecular electrostatics with the linearized Poisson-Boltzmann equation. Biophys J.

[B48] Fogolari F, Esposito G, Viglino P, Molinari H (2001). Molecular mechanics and dynamics of biomolecules using a solvent continuum model. J Comput Chem.

